# Cerebral hemodynamics comparison using transcranial doppler ultrasound and 4D flow MRI

**DOI:** 10.3389/fphys.2023.1198615

**Published:** 2023-05-23

**Authors:** Brandon G. Fico, Kathleen B. Miller, Leonardo A. Rivera-Rivera, Adam T. Corkery, Andrew G. Pearson, Nicole A. Loggie, Anna J. Howery, Howard A. Rowley, Kevin M. Johnson, Sterling C. Johnson, Oliver Wieben, Jill N. Barnes

**Affiliations:** ^1^ Department of Kinesiology, Bruno Balke Biodynamics Laboratory, University of Wisconsin-Madison, Madison, WI, United States; ^2^ Wisconsin Alzheimer’s Disease Research Center, School of Medicine and Public Health, University of Wisconsin-Madison, Madison, WI, United States; ^3^ Department of Medical Physics, School of Medicine and Public Health, University of Wisconsin-Madison, Madison, WI, United States; ^4^ Department of Radiology, School of Medicine and Public Health, University of Wisconsin-Madison, Madison, WI, United States; ^5^ Geriatric Research Education and Clinical Center, William S. Middleton Memorial Veteran’s Hospital, Madison, WI, United States

**Keywords:** aging, cerebral pulsatility, cerebrovascular reactivity, middle cerebral artery, transcranial doppler ultrasound, 4D flow magnetic resonance imaging

## Abstract

**Introduction:** Age-related changes in cerebral hemodynamics are controversial and discrepancies may be due to experimental techniques. As such, the purpose of this study was to compare cerebral hemodynamics measurements of the middle cerebral artery (MCA) between transcranial Doppler ultrasound (TCD) and four-dimensional flow MRI (4D flow MRI).

**Methods:** Twenty young (25 ± 3 years) and 19 older (62 ± 6 years) participants underwent two randomized study visits to evaluate hemodynamics at baseline (normocapnia) and in response to stepped hypercapnia (4% CO_2_, and 6% CO_2_) using TCD and 4D flow MRI. Cerebral hemodynamic measures included MCA velocity, MCA flow, cerebral pulsatility index (PI) and cerebrovascular reactivity to hypercapnia. MCA flow was only assessed using 4D flow MRI.

**Results:** MCA velocity between the TCD and 4D flow MRI methods was positively correlated across the normocapnia and hypercapnia conditions (*r* = 0.262; *p* = 0.004). Additionally, cerebral PI was significantly correlated between TCD and 4D flow MRI across the conditions (*r* = 0.236; *p* = 0.010). However, there was no significant association between MCA velocity using TCD and MCA flow using 4D flow MRI across the conditions (*r* = 0.079; *p* = 0.397). When age-associated differences in cerebrovascular reactivity using conductance were compared using both methodologies, cerebrovascular reactivity was greater in young adults compared to older adults when using 4D flow MRI (2.11 ± 1.68 mL/min/mmHg/mmHg vs. 0.78 ± 1.68 mL/min/mmHg/mmHg; *p* = 0.019), but not with TCD (0.88 ± 1.01 cm/s/mmHg/mmHg vs. 0.68 ± 0.94 cm/s/mmHg/mmHg; *p* = 0.513).

**Conclusion:** Our results demonstrated good agreement between the methods at measuring MCA velocity during normocapnia and in response to hypercapnia, but MCA velocity and MCA flow were not related. In addition, measurements using 4D flow MRI revealed effects of aging on cerebral hemodynamics that were not apparent using TCD.

## Introduction

Cerebral hemodynamics are important biomarkers of brain health (Kleiser and Widder, 1992) and have been shown to be impaired with advancing age. These hemodynamic impairments include a reduction in cerebral blood flow (CBF), even in individuals free of cardiovascular disease (Wu et al., 2016; Alwatban et al., 2021). Another measure of cerebral hemodynamics, cerebral pulsatility, is elevated with advancing age (Alwatban et al., 2021; Fico et al., 2022). When the cerebral vasculature is challenged using elevations in CO_2_ (i.e., hypercapnia), cerebrovascular reactivity is lower in healthy older adults compared to young adults (Barnes et al., 2012; Leoni et al., 2017; Yew et al., 2022). Importantly, these cerebral hemodynamic alterations with age (including lower CBF and elevated cerebral pulsatility) and impaired cerebrovascular reactivity to hypercapnia are associated with Alzheimer’s disease and vascular-related dementias (Richiardi et al., 2015; Rivera-Rivera et al., 2016; Leeuwis et al., 2017; Wolters et al., 2017).

A commonly used method to assess cerebral hemodynamics is transcranial Doppler ultrasound (TCD), which transmits ultrasound waves from the Doppler probe and detects reflected waves from red blood cells passing through the vessel of interest. TCD ultrasound provides rapid, noninvasive measures of cerebral artery blood velocity with excellent temporal resolution and can be utilized by researchers in a variety of settings (Purkayastha and Sorond, 2013). TCD has been used to demonstrate age-related reductions in middle cerebral artery blood velocity and elevations in pulsatility index with advancing age (Alwatban et al., 2021).

Studies using TCD have demonstrated age-related differences in cerebrovascular reactivity based on cerebral artery blood velocity (Bakker et al., 2004; Barnes et al., 2012; Flück et al., 2014). These findings are contrary to studies that show no difference with age (Murrell et al., 2013; Miller et al., 2018), higher values in older adults (Galvin et al., 2010), or sex specific differences (Kastrup et al., 1998). Moreover, when participants are evaluated with multiple methods, such as TCD and magnetic resonance imaging (MRI), these age-related comparisons are inconsistent (Burley et al., 2021a). The discrepancy between reports in age differences in cerebral hemodynamics and cerebrovascular reactivity may be dependent on the sensitivity of the techniques used. A well-known limitation of TCD is the ability to measure blood velocity only, and not blood flow volume rate, because imaging of the arterial diameter or cross-sectional area is not possible. Additionally, the TCD measurement of blood velocity is sensitive to the angle of insonation with error increasing at greater angles (Purkayastha and Sorond, 2013). Thus, if TCD is used to obtain blood velocity, as a surrogate for blood flow, this may result in inconsistent results (Hoiland and Ainslie, 2016b; Brothers and Zhang, 2016).

There are several MRI or neuroimaging methods used to quantify cerebral hemodynamics and cerebrovascular reactivity (Williams et al., 2021). Specifically, blood oxygen level dependent (BOLD) MRI measures the relative levels of oxyhemoglobin and deoxyhemoglobin, which is an indirect measure of CBF and represents activity at the capillary level (Ogawa et al., 1990). Other MRI techniques such as arterial spin labelling (ASL) measures microvascular perfusion (Alsop et al., 2015) and phase contrast angiography (PCA) measures blood flow through the large cerebral vessels such as the middle cerebral artery (MCA). More recently, four-dimensional flow MRI (4D flow MRI or 3D time-resolved PCA) has been used to measure blood velocity in all three spatial dimensions throughout the duration of the cardiac cycle allowing for a direct calculation of blood flow (Markl et al., 2012; Schrauben et al., 2015). However, 4D flow MRI is sensitive to partial volume effects especially when a region of interest is oversized (Tang et al., 1993). Interestingly, MRI based analyses of age-related differences in cerebral hemodynamics and cerebrovascular reactivity also have inconsistent findings (Burley et al., 2021a; Burley et al., 2021b). For example, Burley et al. (2021a) showed that older adults had higher cerebrovascular reactivity to hypercapnia using TCD, but lower cerebrovascular reactivity using BOLD, compared with young adults, further emphasizing the importance of the measurement used to quantify CBF. The discrepancy in findings is likely due to fundamental differences in the MRI methodology and what is quantified. As such, the agreement between the various measurement techniques for cerebral hemodynamics and cerebrovascular reactivity is necessary to be able to compare studies. The commonly used TCD technique may provide various age-related results based on the hypercapnia protocol used (Al-Khazraji et al., 2021) due to its inherent limitation of only capturing blood velocity. Therefore, a more direct method (4D flow MRI) of assessing cerebral blood flow in response hypercapnia may provide further insight into age-related differences.

To our knowledge, no study has directly compared 4D flow MRI with TCD technique to investigate cerebral hemodynamics and cerebrovascular reactivity to hypercapnia in young and older healthy adults. Therefore, the purpose of this study was to compare cerebral hemodynamics of the MCA between TCD and 4D flow MRI in young and older adults at rest and in response to hypercapnia. We hypothesized that the MCA velocity measured using TCD and 4D flow MRI would be correlated and demonstrate good agreement between the two methods. In contrast, we hypothesized that there would be disagreement between the methods when we compared MCA blood velocity using TCD with MCA blood flow volume rate using 4D flow MRI. We also hypothesized that age-associated differences in cerebral hemodynamics and cerebrovascular reactivity would be detected using 4D flow MRI, but not TCD.

## Materials and methods

### Participants

Twenty young (between 18 and 35 years) and nineteen older (between 50 and 68 years) physically active healthy adults participated in the study. Participants were excluded from the study if they had a body mass index (BMI) > 30 kg/m^2^, and if they 1) were current smokers; 2) were diagnosed with hypertension based on the latest guidelines (Whelton et al., 2018) or taking blood pressure medications; 3) presented with a history or evidence of hepatic or renal disease, hematological disease, peripheral vascular disease, stroke, neurovascular disease, cardiovascular disease, diabetes; or 4) had contraindications for a MRI scan (as determined by a health history questionnaire and MRI screening form). All scans were reviewed by a neuroradiologist (HAR) for incidental findings. Of note, data from the participants in this study were also included in our previous publication (Miller et al., 2019). All study procedures were approved by the Institutional Review Board of the University of Wisconsin–Madison and were performed according to the Declaration of Helsinki, including obtaining written informed consent from each participant. This study was registered under ClinicalTrials.gov #NCT02840851.

### Experimental procedures

All procedures were completed at the Bruno Balke Biodynamics Laboratory and the Wisconsin Institutes for Medical Research at the University of Wisconsin–Madison. The study consisted of a screen day visit and two experimental study days. The two experimental study days, with an average of 8 days apart, were randomized and utilized identical protocols for cerebral hemodynamics testing. For each participant, the randomized study days were scheduled for the same time of day to limit the effects of diurnal variation. Premenopausal women were studied in the early follicular phase of their menstrual cycle (or the low-hormone phase of oral contraceptive use) to minimize the influence of ovarian hormone status. Due to the potential influence of diet and exercise on cerebrovascular function, participants were asked to record 3 days of normal dietary intake and exercise prior to their first study day and asked to repeat these prior to their second study day. Prior to the study days, participants were asked to fast for 4 h, to abstain from non-steroidal anti-inflammatory drugs (NSAIDs) for 5 days, avoid nicotine for 2 h, and to abstain from caffeine, exercise, and alcohol for 24 h prior to the visits. Additionally, participants did not take any over the counter medications, vitamins or supplements on the days of the study visits. All tests were conducted in controlled ambient temperature between 20°C and 22°C.

### Screen day visit

Upon arrival to the Bruno Balke Biodynamics Laboratory, height and weight were measured using a standard scale (Seca no. 769, Vogel & Halke, Hamburg, Germany). BMI was calculated as kg/m^2^. Physical activity was determined using a weekly exercise log and physical activity questionnaire (Amireault and Godin, 2015). After 10 min of supine rest, mean arterial pressure (MAP) was taken in triplicate with a non-invasive brachial blood pressure cuff (Datex Ohmeda, GE Healthcare, Fairfield, CT, United States). Participants then had a familiarization session that included TCD set-up with probe location being noted.

### Experimental study day: TCD

We utilized a 2 MHz TCD probe (Spencer Technologies, Redmond, WA, United States) to measure left MCAv (Bishop et al., 1986; Poulin and Robbins, 1996). The 2 MHz probe was placed over the temporal bone above the zygomatic arch between the frontal process and front of the ear with the participants lying supine. The probe was secured using a headband to ensure optimal insonation position and angle throughout the study hypercapnia protocol (Barnes et al., 2012).

### Experimental study day: MRI

Cranial MRI scans were performed at the Wisconsin Institutes for Medical Research using a 3T clinical MRI system (MR750, GE Healthcare, Waukesha, WI, United States) and a 32-channel head coil (Nova Medical Head Coil, Nova Medical, Wilmington, MA, United States) with a gradient strength of 50 mT/m, and a gradient slew rate of 200 mT/m/ms. Left and right middle cerebral artery hemodynamics were assessed using 4D flow Phase Contrast MRI using a 3D radially undersampled sequence that included volumetric, time-resolved PC MRI data with three-directional velocity encoding (PC-VIPR) (Gu et al., 2005; Johnson et al., 2008). Comparisons between TCD and 4D flow MRI utilized the left MCA since TCD measured left MCAv, unless otherwise noted. The imaging parameters were as follows: velocity encoding (Venc) = 80 cm/s, field of view = 220 mm, acquired isotropic spatial resolution = 0.7 mm × 0.7 mm × 0.7 mm, repetition time (TR) = 7.4 ms, echo time (TE) = 2.7 ms, flip angle = 10°, bandwidth = 83.3 kHz, 14,000 projection angles and scan time ∼7 min. Time-resolved velocity and magnitude data were reconstructed offline by retrospectively gating into 20 cardiac phases using temporal interpolation (Jing et al., 2006; Miller et al., 2019).

### Cerebrovascular reactivity to hypercapnia

Hypercapnia trials were performed as previously described in detail using a steady-state, open-circuit technique (Berkenbosch et al., 1989; Miller et al., 2019). Briefly, for both experimental study day visits (study day TCD and study day MRI) participants were in the supine position and fitted with a mask covering their nose and mouth containing a one-way valve to prevent re-breathing (Hans Rudolph Inc., Shawnee, KS, United States). After baseline (using medical grade normocapnic gas), stepwise elevations of 4% and 6% inspired CO_2_, 21% oxygen, and balanced nitrogen were administered. Between stepwise elevations in CO_2_, 2 min were allowed to reach steady state ETCO_2_ prior to collecting cerebral hemodynamics. ETCO_2_ was elevated and maintained constant for approximately 9 min at each level of inspired CO_2_.

### Data analysis

Data from the TCD were collected at 250 Hz and analyzed off-line using signal processing software (WinDaq, DATAQ Instruments, Akron, OH, United States). Cerebral hemodynamics included MCAv and pulsatility index. When using TCD, pulsatility index was calculated as (maximum velocity–minimum velocity)/mean velocity. Beat-by-beat hemodynamic measurements were averaged over 1 min of steady state during normocapnic gas, 4% inspired CO_2,_ and 6% inspired CO_2_. To account for changes in perfusion pressure that may affect MCAv, cerebrovascular reactivity was calculated as the linear relationship between cerebrovascular conductance index (CVCi) calculated as MCAv/MAP and changes in ETCO_2_ during hypercapnia (cm/s/mmHg/mmHg).

The 4D flow MRI scans were also evaluated offline. The scans underwent background phase offset correction, eddy current correction (Schrauben et al., 2015) and automatic phase unwrapping to minimize potential for velocity aliasing (Loecher et al., 2016). Vessel segmentation of the left and right MCA was performed in MATLAB using an in-house tool as previously described for semi-automated cerebrovascular hemodynamic analysis (Schrauben et al., 2015). The MCA was measured at the M1 segment. Measurements included MCAv, blood flow volume rate, and vessel cross sectional area. Hemodynamic measurements were averaged during steady state at normocapnic gas, 4% inspired CO_2_, and 6% inspired CO_2_. When using 4D flow MRI, pulsatility index was calculated as (maximum flow–minimum flow)/mean flow. To account for changes in perfusion pressure that may affect flow, cerebrovascular reactivity was calculated as the linear relationship between cerebrovascular conductance (CVC) calculated as [(blood flow/MAP) x 100] and changes in ETCO_2_ during hypercapnia (mL/min/mmHg/mmHg).

### Statistical analyses

Data analyses were performed using the Statistical Package for the Social Sciences version 28 (SPSS, IBM Corp., Armonk, NY, United States). Statistical differences in participant characteristics were evaluated by the Student’s *t*-tests for unpaired data. Cerebrovascular measurements between young and older adults across conditions were evaluated using two-way (group x condition) analysis of variance. The Greenhouse-Geisser correction of degrees of freedom was used when sphericity assumptions were violated. Significant effects were further analyzed with Bonferroni *post hoc* comparisons. Cerebrovascular reactivity statistical differences between TCD vs. 4D flow MRI were evaluated by the Student’s *t*-tests for paired data and age-related comparisons were evaluated by the Student’s *t*-tests for unpaired data. Associations of interest were analyzed by Pearson correlational analyses and intraclass correlation coefficients were used to determine reproducibility. The intraclass correlation coefficients differs from the Pearson correlations by taking into account differences in the means of the measures being considered (the data are centered and scaled using a pooled mean and standard deviation). The differences between TCD and 4D flow MRI may be minimal at baseline, but the differences may expand at higher levels of CO_2_, due to vessel responses. Using intraclass correlation coefficients we evaluated whether the changes in response to a vasodilatory stimulus agreed between the two methods, at each condition. The statistical procedure proposed by Bland and Altman was used to compare the 2 different methods of TCD vs. 4D flow MRI (Bland and Altman, 1986). Statistical significance was set α priori at *p* < 0.05.

## Results

### Participant characteristics

Participant characteristics are presented in Table 1. There were no group differences between the young and older adults for height, weight, BMI, resting heart rate, metabolic equivalent minutes per week, systolic blood pressure, and MAP. However, the older adults had elevated diastolic blood pressure compared with the young adults.

**TABLE 1:** Characteristics of participants.

Variable	Young adults N = 20	Older adults N = 19	*p*-value
Males/Females (n)	10/10	10/9	
Age (years)	25 ± 3	62 ± 6	**< 0.001**
Height (cm)	173 ± 8	172 ± 9	0.678
Weight (kg)	71 ± 10	70 ± 15	0.841
Body Mass Index (kg/m^2^)	23 ± 2	23 ± 3	0.900
Heart rate at rest (beats per minute)	53 ± 8	55 ± 7	0.488
MET minutes per week	3,259 ± 1839	3,973 ± 2,383	0.300
Systolic blood pressure (mmHg)	120 ± 11	122 ± 11	0.707
Diastolic blood pressure (mmHg)	69 ± 6	74 ± 8	**0.026**
Mean arterial pressure (mmHg)	86 ± 7	90 ± 9	0.120

Data are presented as mean ± standard deviation. MET, metabolic equivalent.

Statistical significance was set α priori at p < 0.05 with significant p-values indicated in bold.

### MCAv comparisons using TCD vs. 4D flow MRI

**FIGURE 1:**
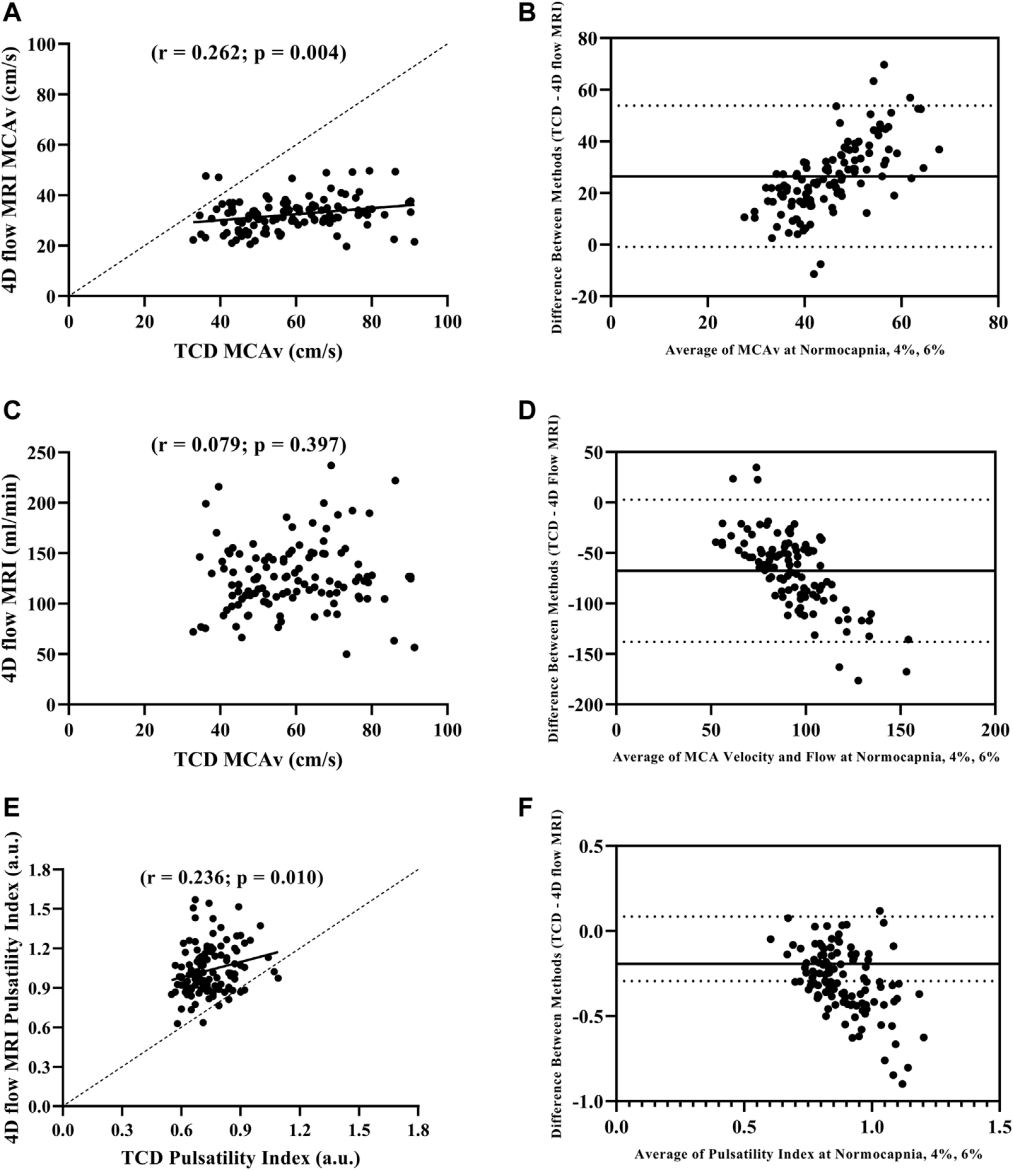
Pearson correlation between MCAv with TCD and 4D flow MRI **(A)**, Bland-Altman plot evaluating agreement between MCAv with TCD and 4D flow MRI **(B)**, Pearson correlation between MCAv with TCD and blood flow volume rate with 4D flow MRI **(C)**, Bland-Altman plot evaluating agreement between MCAv with TCD and blood flow volume rate with 4D flow MRI **(D)**, Pearson correlation between pulsatility index with TCD and 4D flow MRI **(E)**, and Bland-Altman plot evaluating agreement pulsatility index with TCD and 4D flow MRI **(F)** during normocapnia, 4% CO_2_, and 6% CO_2_ including all participants (N = 39).

We first compared MCAv obtained using TCD with MCAv obtained using 4D flow MRI in the left MCA. There was a significant difference in MCAv between the two imaging methods, with higher MCAv being observed with TCD compared with 4D flow MRI across all conditions (all *p* < 0.001; Table 2). Additionally, the percent change in MCAv from normocapnia to 6% CO_2_ was significantly different between TCD and 4D flow MRI (*p* < 0.001; Table 2). Despite these differences, there was a significant intra class correlation coefficient (*r* = 0.329, *p* = 0.016) between the two methods for MCAv, highlighting repeatability. Moreover, although biased, this agreement between MCAv measurements was consistent with the results of the Bland–Altman plot (Figure 1B), and MCAv between the methods was significantly correlated (*r* = 0.262; *p* = 0.004) (Figure 1A).

**TABLE 2:** Cerebral hemodynamics during normocapnia and hypercapnia.

Variable	TCD N = 39	4D flow MRI N = 39	*p*-value
MCAv (cm/s)			
Normocapnia	53 ± 12	31 ± 7	**< 0.001**
4% CO_2_	60 ± 14	32 ± 6	**< 0.001**
6% CO_2_	64 ± 15	33 ± 7	**< 0.001**

MCAv (%Δ)			
Normocapnia to 6% CO_2_	16 ± 10	6 ± 10	**< 0.001**

MCAv (%Δ) vs. MCA Flow (%Δ)			
Normocapnia to 6% CO_2_	16 ± 10	13 ± 10	0.327

Pulsatility Index (a.u.)			
Normocapnia	0.78 ± 0.13	1.07 ± 0.21	**< 0.001**
4% CO_2_	0.73 ± 0.09	1.03 ± 0.17	**< 0.001**
6% CO_2_	0.71 ± 0.09	1.00 ± 0.17	**< 0.001**

Pulsatility Index (%Δ)			
Normocapnia to 6% CO_2_	−9 ± 9	−5 ± 12	0.141

Data are presented as mean ± standard deviation. MCA, middle cerebral artery; MCAv, middle cerebral artery velocity.

Statistical significance was set α priori at p < 0.05 with significant p-values indicated in bold.

### MCAv and MCA flow comparisons using TCD vs. 4D flow MRI

We compared MCAv obtained using TCD with MCA flow obtained using 4D flow MRI. The percent change in MCAv from normocapnia to 6% CO_2_ using TCD vs. the percent change in MCA flow using 4D flow MRI was similar (*p* = 0.327; Table 2). There was no correlation between the left MCAv using TCD and left MCA flow using 4D flow MRI (*r* = 0.079; *p* = 0.397; Figure 1C); however, the Bland-Altman plot suggested moderate agreement (Figure 1D).

### Pulsatility index comparisons using TCD vs. 4D flow MRI

When directly comparing pulsatility index using TCD (calculated from velocity) vs. pulsatility index using 4D flow MRI (calculated from flow), there were significant differences between the methods at normocapnia (*p* < 0.001), and during hypercapnia at 4% CO_2_ (*p* < 0.001) and 6% CO_2_ (*p* < 0.001; Table 2). However, the percent change in pulsatility index from normocapnia to 6% CO_2_ was similar between TCD and 4D flow MRI (*p* = 0.141; Table 2). Additionally, there was a significant intra class correlation coefficient (*r* = 0.345, *p* = 0.012) between the two methods for pulsatility index measurements, highlighting repeatability. Moreover, there was a significant positive correlation between the pulsatility index measurements using TCD vs. 4D flow MRI (*r* = 0.236; *p* = 0.010; Figure 1E), but with weak agreement based on the results of the Bland–Altman plot (Figure 1F).

### Cerebrovascular reactivity comparisons using TCD vs. 4D flow MRI

**FIGURE 2:**
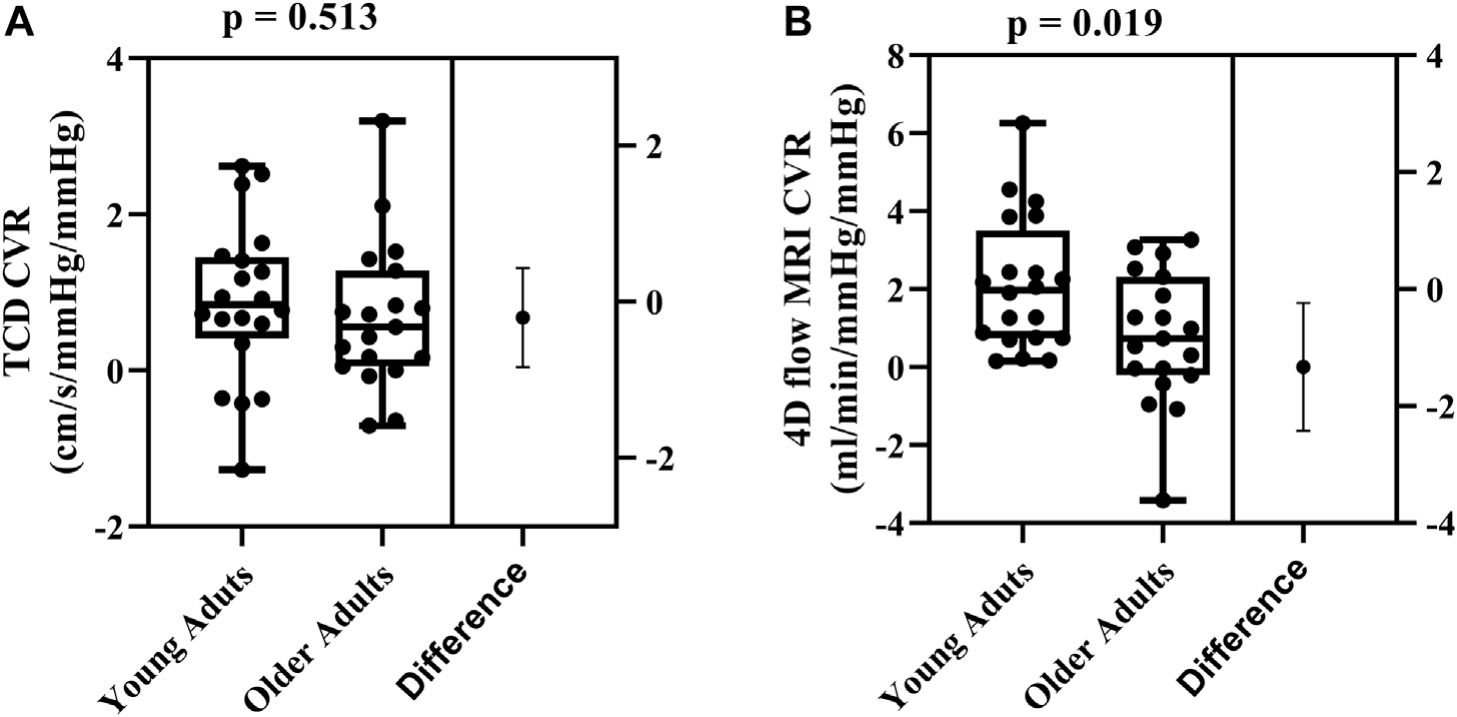
Differences in cerebrovascular reactivity (CVR) between young (N = 20) and older adults (N = 19) evaluated using TCD **(A)** and 4D flow MRI CVR **(B)**. Comparisons were made using Student’s *t*-tests for unpaired data. Data are presented using box and whiskers plots with the boxes indicating where 50% of the data are found, horizontal lines are the medians, and whiskers identify the minimum and maximum values. The mean difference is presented on the far right of each panel with the error bars showing the standard deviation.

Cerebrovascular reactivity values between the two methods were not correlated (*r* = 0.104; *p* = 0.528). Using TCD, there were no age-related differences in cerebrovascular reactivity between young and older adults (0.88 ± 1.01 cm/s/mmHg^2^ vs. 0.68 ± 0.94 cm/s/mmHg^2^, respectively; *p* = 0.513; Figure 2A). In contrast, using 4D flow MRI, cerebrovascular reactivity was higher in young adults compared with older adults (2.11 ± 1.68 mL/min/mmHg/mmHg vs. 0.78 ± 1.68 mL/min/mmHg/mmHg, respectively; *p* = 0.019; Figure 2B).

### Age group comparisons in hemodynamics using TCD vs. 4D flow MRI

**FIGURE 3:**
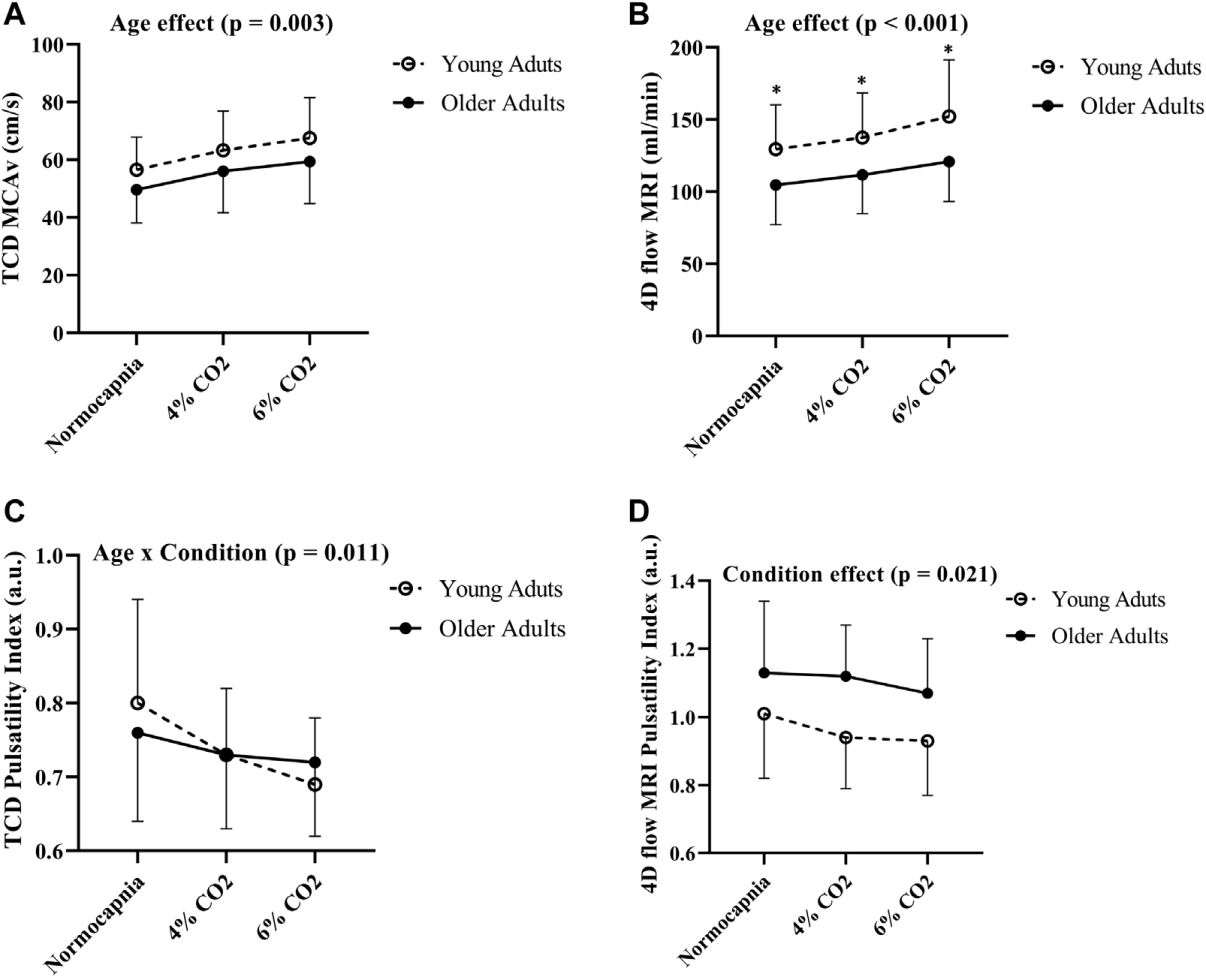
Differences in MCAv **(A)**, blood flow volume rate **(B)**, pulsatility index using TCD **(C)** and pulsatility index using 4D flow MRI **(D)** between young (N = 20) and older adults (N = 19) across all conditions. Age group differences were evaluated using two-way (age group x condition) analysis of variance. Error bars show the standard deviation across the group, * denotes a significant difference (*p* < 0.05) using pairwise comparisons between age groups at each condition.

Comparisons between young and older adults revealed age group differences in MCA blood flow volume rate (using 4D flow MRI) at each condition (*p* < 0.001; Figure 3B). Specifically, young adults had higher blood flow volume rate at normocapnia (*p* = 0.016), 4% CO_2_ (*p* = 0.008), and 6% CO_2_ (*p* = 0.004) as demonstrated in Table 3. There was no difference between young and older adults in MCAv (using TCD) at normocapnia (*p* = 0.069), 4% CO_2_ (*p* = 0.116) and 6% CO_2_ (*p* = 0.080) as demonstrated in Table 3, although there is a significant overall age group effect (*p* = 0.003; Figure 3A). These results highlight that cerebral blood flow measured using 4D flow MRI is more sensitive to age group differences than blood velocity measured using TCD at each condition. Importantly, this is independent of MCA diameter, as there were no differences between the young and older adults MCA diameters at normocapnia, 4% CO_2_ and 6% CO_2_ as demonstrated in Table 3. Additionally, during normocapnia the difference in CVCi (using TCD) between young and older adults did not reach the threshold of significance (Table 3), while CVC (using 4D flow MRI) is higher in young adults compared to older adults (Table 3).

**TABLE 3:** Age comparisons of cerebral hemodynamics during normocapnia and hypercapnia.

Variable	Young adults N = 20	Older adults N = 19	*p*-value
MCAv with TCD (cm/s)			
Normocapnia	57 ± 11	50 ± 12	0.069
4% CO_2_	63 ± 14	56 ± 14	0.116
6% CO_2_	68 ± 14	59 ± 15	0.080

Flow with 4D flow MRI (mL/min)			
Normocapnia	129 ± 31	105 ± 28	**0.016**
4% CO_2_	137 ± 31	112 ± 27	**0.008**
6% CO_2_	152 ± 39	121 ± 28	**0.004**

MCA diameter with 4D flow MRI (mm)			
Normocapnia	2.96 ± 0.14	2.97 ± 0.26	0.886
4% CO_2_	2.95 ± 0.15	2.98 ± 0.27	0.720
6% CO_2_	3.00 ± 0.17	2.99 ± 0.29	0.891

CVCi with TCD (cm/s/mmHg)			
Normocapnia	68 ± 15	59 ± 17	0.085

CVC using 4D flow MRI (mL/min/mmHg)			
Normocapnia	139 ± 35	105 ± 28	**0.001**

Pulsatility Index with TCD (a.u.)			
Normocapnia	0.80 ± 0.14	0.76 ± 0.12	0.249
4% CO_2_	0.73 ± 0.09	0.73 ± 0.10	0.983
6% CO_2_	0.69 ± 0.09	0.72 ± 0.10	0.449

Pulsatility Index with 4D flow MRI (a.u.)			
Normocapnia	1.01 ± 0.19	1.13 ± 0.21	0.061
4% CO_2_	0.94 ± 0.15	1.12 ± 0.15	**0.001**
6% CO_2_	0.93 ± 0.16	1.07 ± 0.16	**0.008**

Data are presented as mean ± standard deviation. CVC, cerebral vascular conductance; CVCi, cerebral vascular conductance index; MCA, middle cerebral artery; MCAv, middle cerebral artery velocity. Statistical significance was set α priori at p < 0.05 with significant p-values indicated in bold.

### Pulsatility index age group comparisons using TCD vs. 4D flow MRI

There were no differences in cerebral pulsatility index (using TCD) between the young and older adults during normocapnia (Table 3). Similarly, cerebral pulsatility index measured using 4D flow MRI was not different between young and older adults during normocapnia (Table 3). There was an interaction effect showing that young adults had a steeper decline in cerebral pulsatility index (measured using TCD) during hypercapnia, compared with the older adults (*p* = 0.011; Figure 3C). Using 4D flow MRI, there was only a condition effect with a similar decrease with hypercapnia between the young and older adults (*p* = 0.021; Figure 3D).

## Discussion

This is the first study to compare cerebral hemodynamics between TCD and 4D flow MRI in healthy adults during normocapnic and hypercapnic conditions. Our results indicate good agreement between the methods for MCAv measured by TCD and MCAv measured by 4D flow MRI. Yet, there was no significant correlation between MCAv measured by TCD and MCA flow measured by 4D flow MRI or cerebrovascular reactivity measured between the two methods. When examining the effect of age, 4D flow MRI was more sensitive to age-related differences in cerebral hemodynamics in response to hypercapnia compared with TCD. Thus, 4D flow MRI may be a useful tool to investigate the impact of age on cerebral hemodynamics.

Our findings that MCAv are in agreement between methods is consistent with recent work demonstrating TCD and 4D flow MRI correlated well for MCAv at normocapnia (Ha et al., 2021). We have further expanded this by comparing MCAv between TCD and 4D flow MRI methods during normocapnia and in response to hypercapnia where cerebral blood flow increases. We demonstrated that MCAv increases with hypercapnia using both TCD and 4D flow MRI; however, the increase in MCAv was greater using TCD when compared to 4D flow MRI. Interestingly, we observed a trend for MCA dilation in response to hypercapnia, which may contribute to the linear bias seen with increasing MCAv between TCD and 4D flow MRI. In other words, we are demonstrating increasing variance between the two methods in response to hypercapnia. It should be noted that there is variability in the literature between the TCD and 4D flow MRI values for MCAv. For example, previous work has demonstrated approximately 30% lower mean velocities with 4D flow MRI compared to TCD (Chang et al., 2011). These differences are likely due to limited insonation angle correction and lack of verification of the same region of interest of vessel segment selected with TCD (Tsuchiya et al., 1991; Hoksbergen et al., 1999). In this context, 4D flow MRI uses velocity encoding multi-directionally inside a 3D volume across time, within a specific vessel segment of interest, allowing for a more averaged MCAv measurement compared to TCD (Markl et al., 2012; Meckel et al., 2013). We speculate that MCAv using TCD is higher than the values reported using 4D flow MRI because the sample volume (region of interest) with TCD is narrower and captures the velocity in the middle of the vessel where the velocity is the highest due to laminar flow. While 4D flow MRI captures and averages the MCAv throughout the entire vessel (average velocity across the voxels included in the region of interest), this lowers the average MCAv compared with TCD. In addition, TCD is highly operator dependent and could limit reproducibility (Baumgartner et al., 1994), while 4D flow MRI has been demonstrated to have good test-retest reliability, multicenter reproducibility, and interobserver agreement (Wen et al., 2019).

The benefit of using 4D flow MRI is that assessments of blood flow volume rate (rather than only blood velocity) can be made because of the cross-sectional area data (Markl et al., 2012). In addition, 4D flow MRI allows for simultaneous segmentation of multiple cerebral vessels (Markl et al., 2012; Rivera-Rivera et al., 2016; Miller et al., 2019). In the present study, we were interested in comparing cerebrovascular hemodynamics using blood flow volume rate with 4D flow MRI vs. blood velocity with TCD. Importantly, we observed similar increases in blood flow volume rate and blood velocity in response to hypercapnia. We also observed moderate agreement between blood flow volume rate using 4D flow MRI and blood velocity using TCD. Taken together, our results indicate MCAv can be used as an indicator for blood flow responses to hypercapnia in healthy young and older adults.

In the present study, we were interested in comparing changes in cerebral pulsatility in response to hypercapnia with both imaging modalities. We observed similar decreases in cerebral pulsatility with TCD and 4D flow MRI. We also evaluated age-related differences between the methods. For example, cerebral pulsatility index decreased similarly in response to hypercapnia in the young and older participants with 4D flow MRI, yet we reported a steeper decrease in young adults compared with older adults when using TCD. Our results are in agreement with previous research demonstrating cerebral pulsatility decreases in response to mild hypercapnia using 4.5% CO_2_ (DuBose et al., 2022). Importantly, we demonstrated TCD provided agreement and similar age-related results for MCAv when compared with the more advanced measurement technique of 4D flow MRI.

Previous work has demonstrated variability in age-related findings of cerebrovascular reactivity, particularly when comparing young and older adults (Peng et al., 2018; Miller et al., 2019). When investigating age-related differences using two methods TCD vs. MRI (BOLD; PCA), Burley et al. (2021a) reported higher cerebrovascular reactivity using TCD in older adults compared to younger adults, but the opposite results when using BOLD MRI, with younger adults having greater cerebrovascular reactivity than the older adults. Our results showed age-related differences in cerebrovascular reactivity between young and older adults, when using 4D flow MRI, but not with TCD. One possible reason for these differences is based on methods because TCD relies on the assumption that the MCA diameter does not change during hypercapnia. Based on this assumption, MCAv would be a reasonable surrogate for MCA flow; however, this assumption is still debated (Hoiland and Ainslie, 2016a; Brothers and Zhang, 2016). Although the present study did not show a correlation between MCAv and MCA flow in response to hypercapnia, it is possible that age may influence MCA cross-sectional area (Miller et al., 2019), and explain why the results regarding age differences were linked to the specific methodology. The discrepancies in the previous research may be explained by potential MCA cross sectional area changes (Verbree et al., 2014), with evidence that percent change increases in MCA cross-sectional area with hypercapnia are attenuated in older adults compared to young adults (Coverdale et al., 2017; Miller et al., 2019). Because of the user-dependent nature of TCD, it is possible that the lack of age-related differences in cerebrovascular reactivity in this study was related to sample size. In order to detect significant age differences in cerebrovascular reactivity using TCD, a greater sample size may be necessary.

Cerebral hemodynamics and cerebrovascular reactivity are emerging biomarkers for a range of conditions, including Alzheimer’s disease (Roher et al., 2012; Rivera-Rivera et al., 2017; Sur et al., 2020). Because there is currently no “gold standard” for cerebral hemodynamic measurements, the purpose of this project was to compare two common methods to assess cerebral hemodynamics. 4D flow MRI is a newer method of assessing vessel-specific flow parameters compared with TCD and offers many benefits including the ability to measure cerebral hemodynamics in multiple vessels simultaneously. There are other benefits to TCD compared with MRI, including 1) portability; 2) that it is comparatively cost-effective; and 3) TCD can be less invasive/intrusive than MRI procedures (Ghorbani et al., 2010). In the present study TCD was able to distinguish a greater decrease in cerebral pulsatility in the young adults compared to older adults. In addition, TCD has excellent temporal resolution for rapid physiological responses, which are not yet available with MRI. Additionally, TCD has clinical applications such as diagnosing cerebral vasospasm, guiding transfusion therapy, and providing preoperative evaluation for patients with cerebrovascular disease (Kincaid, 2008). Our findings may help future studies in determining the methodology that provides the best approach for the intended research question.

This study is not without limitations. For example, it was not possible to capture TCD and 4D flow MRI hemodynamics on the same day. Therefore, some variability between the measurements would be expected due to individual variation in hemodynamics (Rickards and Tzeng, 2014; Ismaili et al., 2018). A strength of the study is we attempted to minimize day-to-day variability by replicating the same procedures for each visit within a relatively short time frame with strict diet and exercise routines being duplicated. Specifically, we had participants record 3 days of normal dietary intake and exercise prior to their first study day and asked them to repeat these prior to their second study day. As mentioned, an inherent limitation when comparing TCD vs. 4D flow MRI is TCD relies on the assumption that blood velocity is a surrogate for blood flow and may not be ideal in experimental set-ups that elicit MCA dilation. Another limitation is that the instrumentation between visits was not identical due to constraints with MRI compatibility. Additionally, we did not examine relationships between TCD or 4D flow MRI with BOLD-based cerebrovascular reactivity measurements, which are commonly used. The goal of this project was to investigate MCA vessel-specific velocity and flow responses, but future studies could compare 4D flow MRI with BOLD-based cerebrovascular reactivity assessments. A final limitation is that the older adults included in this study were healthy and met or exceeded published physical activity guidelines, which may limit the translation of this study to the general population who are sedentary, may have vascular risk factors or established disease.

In conclusion, our study demonstrated good agreement between the two imaging methods for MCAv. There was no correlation between MCAv measured by TCD and MCA flow measured by 4D flow MRI or cerebrovascular reactivity measured between the two methods. When performing age-related comparisons, 4D flow MRI was more sensitive to age-related differences between young and older adults. Due to these capabilities when using 4D flow MRI, we were able to demonstrate lower blood flow volume rate and CVC in older adults when compared to younger adults, which was not possible with TCD. Additionally, 4D flow MRI provides clinical utility such as characterizing changes in cerebrovascular hemodynamics with Alzheimer’s disease (Rivera-Rivera et al., 2016; Berman et al., 2017; Rivera-Rivera et al., 2017). Future studies could compare cerebral hemodynamics and cerebrovascular reactivity between 4D flow MRI and other MRI techniques (e.g., BOLD, ASL) to help determine the appropriate methodology for the proposed research questions.

## Data Availability

The raw data supporting the conclusion of this article will be made available by the authors, without undue reservation.
